# Sexual network and detection of anogenital human papillomavirus in a community cohort of men who have sex with men in Taiwan

**DOI:** 10.1371/journal.pone.0216784

**Published:** 2019-05-10

**Authors:** Carol Strong, Yi-Fang Yu, Huachun Zou, Wen-Wei Ku, Chia-Wen Lee, Nai-Ying Ko

**Affiliations:** 1 Department of Public Health, National Cheng Kung University Hospital, College of Medicine, National Cheng Kung University, Tainan, Taiwan; 2 School of Public Health (Shenzhen), Sun Yat-sen University, Shenzhen, China; 3 School of Public Health, Sun Yat-sen University, Guangzhou, China; 4 Kirby Institute, University of New South Wales, Sydney, Australia; 5 Division of Infectious Diseases, Taipei Veterans General Hospital, Taipei, Taiwan; 6 Fengshan Lee Chia Wen Urologic Clinic, Kaohsiung, Taiwan; 7 Department of Nursing, National Cheng Kung University and Hospital, College of Medicine, National Cheng Kung University, Tainan, Taiwan; 8 Institute of Allied Health Sciences, College of Medicine, National Cheng Kung University, Tainan, Taiwan; The Chinese University of Hong Kong, HONG KONG

## Abstract

**Objectives:**

We examined the association between anogenital human papillomavirus (HPV) infection and sexual networks in men who have sex with men (MSM).

**Methods:**

A total of 253 MSM, 20 years of age and older, were recruited from the community in Southern Taiwan in 2015–2016. At baseline and at each follow-up visit, MSM were screened for HPV to identify 37 HPV genotypes. At the six-month follow-up, MSM were asked to fill out an egocentric network assessment and to report the last five persons with whom they had sex regarding the characteristics of sexual behavior with each network member.

**Results:**

A total of 182 participants (71.9%) returned for the follow-up and one third had at least one HPV type detected. A higher level of bridging network position calculated by the level of constraints in the network was significantly less likely to have HPV detection at the anal site. A high level of concurrency was associated with penile HPV detection (AOR = 3.16, 95% CI = 1.01–9.86).

**Conclusions:**

Identifying network-related characteristics can advance our understanding of high-risk populations and for prioritizing HPV vaccine recommendations.

## Introduction

Human Papillomavirus (HPV) is significantly associated with cancers and diseases in both men and women. HPV is responsible not only for cervical cancer but also for 90% of anal cancers, 40% of penile cancers, and 12% of oral and pharyngeal cancers [1]. Genital HPV infection is most commonly transmitted through sexual intercourse and other intimate contact, such as oral-genital or genital-genital sexual practices [2]. Some HPV infections are clinically insignificant and asymptomatic; however, when symptoms occur, they include warts, dysplasia, or frank malignancy [1].

The prevalence data for HPV infection in men varies by continent, country, ethnicity, recruitment method, age, HIV history, sexual orientation, and sample size [3]. Several studies have shown high prevalence of HPV infection in men who have sex with men (MSM). One study in the U.S. showed a high prevalence of HPV infection in a sample of MSM 16–30 years of age [4]. As high as 70% of young MSM had incurred HPV infection detected and HPV was more likely to be detected if these MSM had a higher number of lifetime male receptive anal sex partners [4].

A high prevalence of HPV infection was also reported in European countries, but it was not as high in Australia. Two studies in European countries recruited MSM from STD clinics and found anal HPV infection prevalence around 69%–77% [5–7]. In Australia, the prevalence of anal HPV infection among young MSM ages 16–20 appears much higher for those with more than three receptive anal sex partners (47.3%) than among those reporting no prior receptive anal sex (10.0%) [8].

A cost-effectiveness study found that providing HPV vaccine to 12-year-old girls can be cost-effective in preventing many cases of cervical cancer and death [9]; however, more debate has surrounded whether it is cost-effective to vaccinate males as well [10]. Regardless, a global trend in health policies related to HPV vaccination is moving in the direction of vaccinating males, such as in Australia, where a free vaccination program was enacted in 2013 [11].

MSM is a highly neglected population in the current recommendation of girls-only vaccination programs in most countries, including Taiwan. Although the disease burden of HPV is higher in women than in men, by continuing to center HPV attention on women, a social environment was created that potentially reinforced the stigma of HPV infection, which may hinder HPV prevention efforts [12].

Under the circumstances in several countries in which resources are limited and the cost for HPV vaccines are still high, prioritizing high-risk male populations for raising awareness and updating of vaccinations may help reduce the burden associated with HPV. Therefore, it is important to identify target populations that are at higher risk for HPV infection for interventions or policy enactment.

In the current literature, risk factors of HPV infection often include individual-level factors, such as having a higher number of lifetime receptive anal sex occurrences between male partners [4, 8]. Using a social network approach to identify network-related characteristics can further incorporate information regarding the individuals’ position in their sexual network to advance our understanding regarding high-risk populations.

The concept of social networks, which describes the existence of social ties, has been used to understand the spread of sexually transmitted infections (STIs). Social networks among MSM function as an immediate social environment that shapes network members’ shared behavioral norms and high-risk behavioral characteristics [13]. Social network attributes—such as structure, density of the sexual network indicated by the proportion of connections in a sexual network, and how popular that person is within a sexual network—are sometimes stronger factors responsible for HIV incidence in MSM community populations than for individual behaviors [13, 14]. Given the rarity of HPV screening compared to other STIs, no research has been published regarding sexual networks and HPV infection. Although no studies have examined the association of HPV and sexual networks, other STIs, especially HIV, have received much attention in the public health literature [15–18].

HPV infection provides an interesting platform to study the complexity of social and sexual networks. Unlike HIV, people who are detected with HPV subtypes at one point does not mean that they will still be detected in six months because of effective immunity [19, 20]. In any population, the prevalence of HPV is much higher than HIV [3]. HPV has several subtypes and not all subtypes affect men in the same way, which adds another level of complexity. High-risk types are associated with cancers, while low-risk types, although non-oncogenic, are associated with genital warts [21, 22]. Low-risk types 6 and 11 are associated with 85%-95% of genital warts [21]. HPV screenings are not included in routine STI checkups, partly due to the higher cost. Examining sexual networks in relation to HPV infection not only helps identify high-risk populations that might need to be prioritized for vaccination, but also adds to our understanding of HPV infection.

In this study, we used HPV infection as a vehicle to study the association between sexual networks and STI transmission in a community cohort of MSM. The aims include: (a) to collect baseline sexual network characteristics via egocentric network assessment, and (b) to identify network characteristics associated with anal and penile HPV detection in a community cohort of adult MSM. Studies that examined prevalence and risk factors for HPV infection often report anal and penile sites separately because potential risk factors may predict one over the other [8, 23]. An egocentric approach centers on a focal actor (ego) and that individual’s network contacts (alters), even though alters might not be the main study participants [24]. Specifically, we accomplished the following: (a) described the profile of the baseline sexual network characteristics of a cohort of MSM recruited in Taiwan, (b) examined the sexual relationships between egos and alters, and (c) identified network-related risk factors associated with anal and penile HPV detection.

## Materials and methods

### Participants and procedures

A total of 253 MSM, 20 years of age and older, were recruited from the community. To be eligible, participants needed to have had sex with another male—mutual masturbation, oral or anal sex—and be willing to give consent to participate in the study. The major recruitment channel was through LGBTQ community health centers in Southern Taiwan. Study participants received three HPV screening tests in the 1-year frame (baseline, 6^th^ and 12^th^ month) and then annually in the 2^nd^ and 3^rd^ year. For each visit, MSM received HPV screening, including anal and penile swabs, and a self-administered paper-and-pencil survey assessing factors related to HPV infection. HPV DNA testing and genotyping was performed by polymerase chain reaction. In the follow-up surveys, we included a complete sexual network assessment to generate names and interpret the network relations. For this paper, we used data from the six-month follow-up collected during March to December 2016. A total of 182 participants (71.9%) returned for the six-month follow-up. The study was approved by the Ethics Committee of the National Cheng Kung University Hospital (reference number: A-BR-103-075). All participants received clear information about the study, fully understood the study purpose, and signed a written informed consent.

### HPV detection

Study nurses obtained a sample from the anal and penile sites for all participants. Samples were processed in labs: (a) first via ROCHE cobas 4800 HPV Amplification Detection Kit, a qualitative multiplex assay detecting HPV in patient specimens. (b) If results showed positive, samples were further processed by ROCHE LINEAR ARRAY HPV genotyping test, a qualitative test that detects 21 high-risk (16, 18, 26, 31, 33, 35, 39, 45, 51, 52, 53, 56, 58, 59, 66, 67, 68, 69, 70, 73 [MM9], 82 [MM4]) and 16 low-risk HPV genotypes (6, 11, 40, 42, 54, 55, 61, 62, 64, 71 [CP8061], 72, 81 [CP8304], 83 [MM7], 84 [MM8], 82v, 89).

### Measures of network-related parameters

#### Name generator and interpreters

Name generator was used to elicit names of sexual network members. We asked participants to list the last five persons with whom they had had sex. The number of sexual partners was capped at five considering time constraints and respondent burden, which is also the number suggested for egocentric network data collection [25]. Under each set of five names, participants were asked to report their sexual network members’ information, including sociodemographics, whether they were the receptive partner during anal sex with each sexual network member, types of relation when he and his sexual partner first had sex, with the following options: they have never met before; they have known each other for a while (acquaintance); they were friends but never dated; they were dating but not in a relationship; they were partners; sex was exchanged for money, drugs, housing or other benefit. We distinguished the types of relation when they first had sex based on whether they were acquainted or not and grouped “they have never met before” and “sex was exchanged for money, drugs, housing, or other benefit”together as the more risky type of relation versus the rest of the options (i.e., acquaintance/friends/been dating/partners).

We asked participants regarding time at first and last sex with each sexual network member (e.g., oral, anal or vaginal sex), the frequency of sex and using a condom when they had sex, and substance use during sex. We also asked participants whether each sexual network member was HIV positive on a scale of definitely yes, very likely yes, not sure, very likely not, and definitely not. We dichotomized the answers into definitely yes/very likely yes vs. not sure/very likely not/definitely not.

#### Concurrency

Concurrency was determined via the date of the first and last time they had sex with each alter. It was categorized as having no overlap partners, had two or more alters overlapped in any time period.

#### Bridging

We asked participants to report whether they thought any two of their sex partners may have had sex previously on a scale of definitely yes, very likely, not sure, very likely not, or definitely not. We created a tie between two alters indicating potential sex partners if they answered definitely yes or very likely. Structural constraint was calculated with igraph package in R software [26]. The level of bridging network position was based on the constraint metric in which the constraint score is higher if ego has fewer contacts, or if ego has more redundant/mutually stronger, related contacts [26, 27]. Higher constraint scores reflected a lower level of bridging. Bridging status was categorized as high constraint (c) (c ≦ 0.25), medium constraint (0.25 < c ≦ 0.5), and low constraint (c > 0.5).

### Analysis

We described the characteristics and sociodemographics of egos and their alters. All relevant data are within the paper and its Supporting Information files (S1–S3 Tables). For each ego-network, we calculated a proportion for each risk behavior to occur between ego and each alter. Outcomes included HPV detection at penile or anal site separately, and any-, high-, or low-risk types. Risk factors that showed marginal statistical significance or statistical significance in bivariate analysis were included in multivariable regression. Multivariable logistic regression analysis was used to estimate network-related factors associated with any-risk type of HPV detection; multivariable multivariate probit regression was used for high- and low-risk types of HPV detection.

## Results

At the six-month follow-up, among 182 MSM from the community (71.9%), 178 participants had valid anal HPV results and 171 participants had valid penile HPV results. Almost half of men (44.4%) had at least one HPV type detected (Table 1). HPV detection was more likely to be detected at the anal site than the penile site (39.0% vs. 16.5%, *p* < .05). For the anal site, 25.3% had at least one high-risk type detected and 22.0% had one low-risk type. For the penile site, 10.4% had at least one high-risk type and 7.7% had one low-risk type.

**Table 1:** Sample characteristics: Sociodemographics of ego and alter, and ego-network characteristics.

				Ego (N = 182)	Alter (N = 631)	Ego-network
				N (%)	N (%)	Mean (SD)
**Age**						
	<25			50 (27.5)	164 (26.0)	-
	25–34			108 (59.3)	351 (55.6)	-
	35–44			21 (11.5)	90 (14.3)	-
	45+			3 (1.7)	23 (3.7)	-
**Highest level of education**						-
	Elementary school		0 (0)	1 (0.2)	-
	Higher school		17 (9.3)	85 (13.5)	-
	University		121 (66.5)	439 (69.6)	-
	Graduate school		44 (24.2)	95 (15.1)	-
**Sexually transmitted infections (STI)**						
	WAVE 1+2: lifetime STI (gonorrhea, genital warts, syphilis, genital herpes, *molluscum contagiosum*)			45 (24.7)	-	-
	WAVE 2: in the past 6 month STI (gonorrhea, genital warts, syphilis, genital herpes, *molluscum contagiosum*)			16 (8.8)	-	-
	**Ego HIV status**					
			WAVE 1+2: lifetime self-reported positive	9 (5.0)	-	-
			WAVE 2: in the past 6 month self-reported positive	5 (2.8)	-	-
	**Alter HIV status**					
			Confirmed yes	-	13 (2.1)	-
			Very likely yes	-	3 (0.5)	-
			Not sure	-	232 (36.8)	-
			Very likely not	-	141 (22.4)	-
			Confirmed no	-	242 (38.4)	-
	**HPV**					
			Anal			
			High-risk type	46 (25.3)	-	-
			Low-risk type	40 (22.0)	-	-
			Any risk type	71 (39.0)	-	-
			Penile			
			High-risk type	19 (10.4)	-	-
			Low-risk type	14 (7.7)	-	-
			Any risk type	30 (16.5)	-	-
	**Numbers of HPV type (out of 37)**				
			1	50 (27.5)	-	-
			2	21 (11.5)	-	-
			3	8 (4.4)	-	-
			4	6 (3.3)	-	-
			5	1 (0.6)	-	-
**Measure of each egos' level of bridging**^**a**^						
			Low bridging (c > 0.5)	68 (37.4)	-	-
			Moderate bridging (0.25 < c ≦ 0.5)	48 (26.4)	-	-
			High bridging (c ≦ 0.25)	66 (36.3)	-	-
**Concurrency: Ego having sexual partnerships that overlap in time**						
			Not overlap	99 (54.4)	-	-
			Having two sexual partnerships in any time period	29 (15.9)	-	-
			Having more than two sexual partnerships in any time period	54 (29.7)	-	-
**Proportion of each behavior in the ego-network**						
	**Sexual behavior**				
			Ego as the receptive partner during anal sex	-	-	.40 (.40)
			Types of relation when ego and alter first have sex (never met before/sex was exchanged for money or drug vs. acquaintance/friends/been dating/partners)	-	-	.42 (.40)
			Ego is ≧1 age category younger than the alter^b^	-	-	.23(.34)
	**Substance use**				
			Alter being a smoker in the period when ego and alter are in a sexual relationship	-	-	.13 (.24)
			Using alcohol when ego and alter have sex	-	-	.20 (.34)
	**Infection**				
			Alters being HIV positive (definitely yes/very likely yes vs. not sure/very likely not/definitely not)	-	-	.03 (.12)

^a^High bridging means low constraint score.

^b^Age category difference is based on the following age categorical variables of the sexual partners in the sexual network inventory: below 25, 25–35, 35–45, above 45 years old.

**Fig 1:**
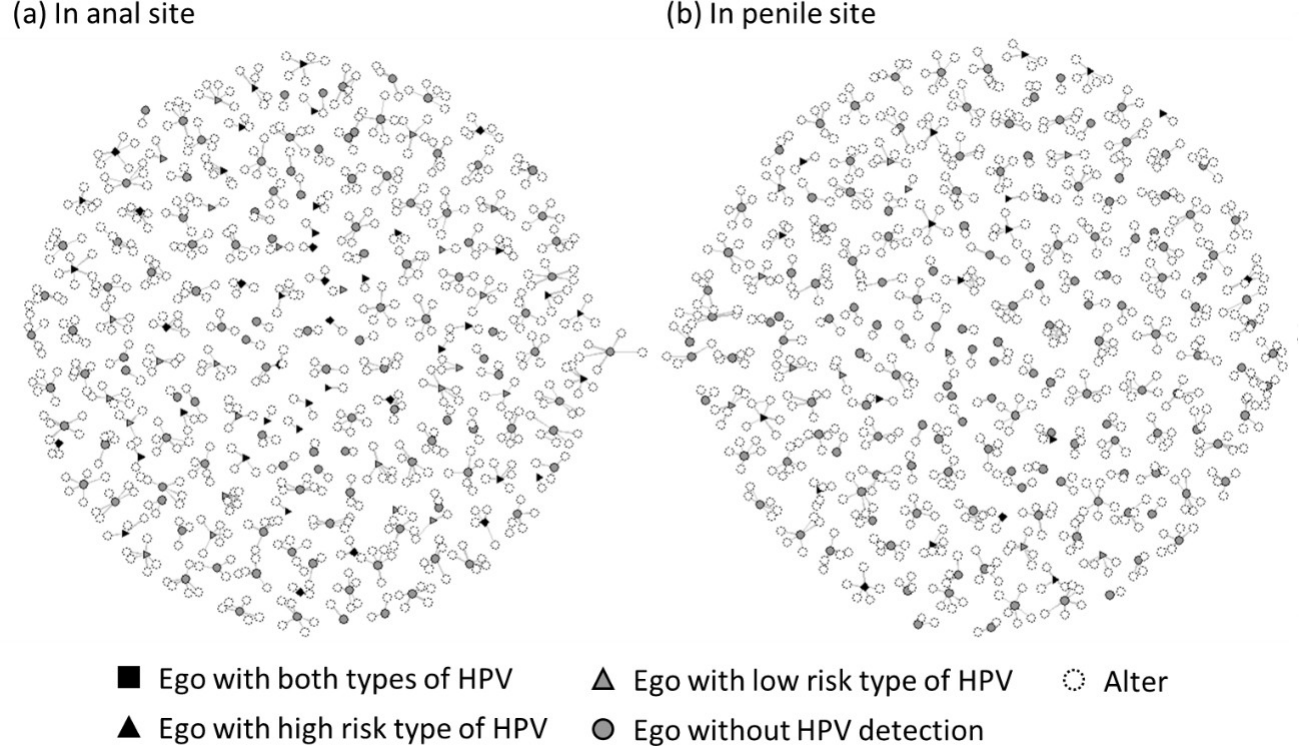
HPV detection denoted on the network graph.

Out of 182 egos, 631 alters were reported. Almost half of the participants reported five alters (49%), 14% reported four or three alters, and 36% reported 1 or 2 alters. In Fig 1, we denote HPV detection on the sexual network graph. Most alters were recent sex partners. When calculating the time between the last sex with any alter and the 2^nd^ wave, 77.5% were within one year, 10.1% were between 1 to 2 years, and 9.8% were between 2 to 5 years.

In Table 1, we present sociodemographics and ego-network characteristics among egos, alters, and the ego-network. For egos, the majority was rather young; most of the egos were below age 35 (86.8%) and had at least a college degree (90.7%). About one fourth had been diagnosed with an STI in their lifetime, including gonorrhea, genital warts, syphilis, genital herpes, or *molluscum cotagiosum*, and 5% had been diagnosed with HIV. The majority of alters were also below age 35 (81.6%) and had a college degree (84.7%). When egos were asked to report their alter’s HIV status, 2% reported a confirmed positive status, about 60% were not sure about their status, and 38% thought that their partner was a confirmed negative.

For concurrency, 54% of the egos had no overlap partners, 15% had two alters overlapped, and 30% had more than two. On average, in 40% of the ego network, the ego was the receptive partner during anal sex. When the ego and alter first had sex, 42% had either never met before or had exchanged sex for money, drugs, housing, or other benefit. Twenty percent used alcohol when they had sex. In 3% of the ego network, alters were definitely or very likely to be HIV positive.

In Table 2, we present multivariable logistic regression results for network-related factors associated with any-risk type of HPV detection. At the anal site, having a moderate bridging level was associated with a 70% risk reduction of HPV detection compared to the low-bridging group (AOR = 0.30, 95% CI = 0.12–0.78); the high bridging group was 63% less likely to have any-risk type of HPV detection compared to the low-bridging group (AOR = 0.38, 95% CI = 0.16–0.92). For the penile site, a high level of concurrency was significantly associated with HPV detection (AOR = 3.16, 95% CI = 1.01–9.86).

**Table 2:** Network-related factors associated with any-risk type of HPV detection (multivariable logistic regression).

			Any-risk type	
			Anal, OR(95%CI) (N = 178)	Penile, OR(95%CI) (N = 171)
**Measure of each egos' level of bridging**^**a**^				
		Low bridging (c > 0.5)	ref	ref
		Moderate bridging (0.25 < c ≦ 0.5)	0.30* (0.12,0.78)	0.78 (0.22,2.80)
		High bridging (c ≦ 0.25)	0.38* (0.16,0.92)	0.86 (0.25,2.98)
**Concurrency: Ego having sexual partnerships that overlap in time**				
		Not overlap	ref	ref
		Having two sexual partnerships in a time period	1.46 (0.55,3.89)	0.9 (0.21,3.83)
		Having more than two sexual partnerships in a time period	1.71 (0.70,4.15)	3.16* (1.01,9.86)
**Proportion of each behavior in the ego-network**				
	**Sexual behavior**			
		Ego as the receptive partner during anal sex	1.88 (0.81,4.34)	1.05 (0.35,3.19)
		Types of relation when ego and alter first have sex (never met before/sex was exchanged for money or drug vs. acquaintance/friends/been dating/partners)	1.48 (0.65,3.40)	1.05 (0.35,3.14)
		Ego is ≧1 age category younger than the alter^b^	0.88 (0.33,2.33)	0.69 (0.18,2.60)
	**Substance use**			
		Alter being a smoker in the period when ego and alter are in a sexual relationship (yes vs. no/not sure)	2.43 (0.59,9.95)	1.05 (0.17,6.52)
		Using alcohol when ego and alter have sex (yes vs. no)	1.07 (0.41,2.83)	2.21 (0.66,7.38)
	**Infection**			
		Alters being HIV positive (definitely yes/very likely yes vs. not sure/very likely not/definitely not)	3.03 (0.14,65.64)	0.36 (0.00,53.11)

* p < .05, Ref: reference group.

^a^High bridging means low constraint score.

^b^Age category difference is based on the following age categorical variables of the sexual partners in the sexual network inventory: below 25, 25–35, 35–45, above 45 years old.

In Table 3, we further separated high- and low-risk types of HPV detection. Multivariable multivariate probit regression analysis showed that the high-risk type of HPV detection at the anal site was associated with high levels of bridging (AOR = 0.51, 95% CI = 0.28–0.93) and that a higher proportion of egos were the receptive partners during anal sex (AOR = 1.80, 95% CI = 1.04–3.11). A low-risk type of HPV detection at the anal site was associated with a riskier type of relation when the ego and alter first had sex, that is, had never met before or had exchanged sex for money, drugs, housing, or other benefit (AOR = 2.44, 95% CI = 1.36–4.38), and alters were HIV positive (AOR = 13.83, 95% CI = 1.59–120.40). In multivariable analysis, no risk factors appeared significant for penile site detection.

**Table 3:** Network-related factors associated with high-risk or low-risk type of HPV detection (multivariable multivariate probit regression).

			Anal, OR(95%CI, N = 178)		Penile, OR(95%CI, N = 171)	
			High-risk type of HPV	Low-risk type of HPV	High-risk type of HPV	Low-risk type of HPV
**Measure of each egos' level of bridging**^**a**^						
		Low bridging (c > 0.5)	ref	ref	ref	ref
		Moderate bridging (0.25 < c ≦ 0.5)	0.62 (0.34,1.13)	0.53 (0.27,1.02)	0.69 (0.30,1.57)	1.35 (0.53,3.47)
		High bridging (c ≦ 0.25)	0.51* (0.28,0.93)	0.72 (0.39,1.31)	0.81 (0.37,1.79)	1.43 (0.55,3.68)
**Concurrency: Ego having sexual partnerships that overlap in time**						
		Not overlap	ref	ref	ref	ref
		Having two sexual partnerships in a time period	1.19 (0.63,2.28)	1.65 (0.85,3.22)	1.08 (0.45,2.61)	0.68 (0.21,2.16)
		Having more than two sexual partnerships in a time period	1.21 (0.67,2.17)	1.32 (0.71,2.44)	1.90 (0.88,4.10)	1.60 (0.71,3.61)
**Proportion of each behavior in the ego-network**						
	**Sexual behavior**					
		Ego as the receptive partner during anal sex	1.80* (1.04,3.11)	1.00 (0.55,1.80)	1.60 (0.78,3.28)	0.64 (0.25,1.59)
		Types of relation when ego and alter first have sex (never met before/sex was exchanged for money or drug vs. acquaintance/friends/been dating/partners)	0.80 (0.46,1.37)	2.44** (1.36,4.38)	1.19 (0.61,2.35)	0.68 (0.29,1.56)
		Ego is ≧1 age category younger than the alter^b^	0.85 (0.45,1.62)	1.69 (0.85,3.35)	1.07 (0.49,2.35)	0.19 (0.03,1.25)
	**Substance use**					
		Alter being a smoker in the period when ego and alter are in a sexual relationship (yes vs. no/not sure)	0.96 (0.40,2.34)	2.11 (0.87,5.11)	0.92 (0.28,3.05)	0.63 (0.11,3.44)
		Using alcohol when ego and alter have sex (yes vs. no)	0.77 (0.41,1.45)	1.47 (0.75,2.89)	0.93 (0.39,2.19)	2.02 (0.82,4.97)
	**Infection**					
		Alters being HIV positive (definitely yes/very likely yes vs. not sure/very likely not/definitely not)	0.21 (0.02,2.04)	13.83* (1.59,120.40)	NA	2.70 (0.11,65.64)

* p < .05

**p < .01 , Ref: reference group.

^a^High bridging means low constraint score.

^b^Age category difference is based on the following age categorical variables of the sexual partners in the sexual network inventory: below 25, 25–35, 35–45, above 45 years old.

## Discussion

To the best of our knowledge, this is the first study to examine the association between network-related factors in association with HPV infection. The study demonstrated sexual network-related risk factors in association with anogenital HPV detection in a community sample of MSM. Using egocentric network analysis to quantify sexual behaviors and characteristics between study participants and their most recent five sex partners, a few network-related factors were in line with previous understanding of STI transmission, such as whether high levels of concurrency were significantly associated with penile HPV detection. Based on whether two previous sex partners might potentially have a sexual relationship, a higher level of bridging was significantly less likely to have HPV detection at the anal site. Other commonly reported risk factors in HIV and STI research were found to be associated with either high- or low-risk types of anal HPV detection; for instance, a study participant being the receptive partner during anal sex, having HIV-positive sex partners, or alters with whom the first sex between them was more likely when they had just met or had exchanged sex for drugs or other benefits. Our study provides information to identify MSM populations at high risk for HPV infection.

The often asymptomatic and undiagnosed nature of HPV infection offers an opportunity to examine the association of social links on sexual behaviors and sexual health. Instead of asking participants to report a proportion of risky sexual behaviors, the egocentric network approach requires participants to recall details for behaviors and characteristics for each partner. A large number of sexual network studies related to STI and MSM were conducted in the context of Black MSM in the U.S. [28]. A review indicated that the studies tended to report HIV risk as being associated with high levels of assortative mixing by race and disassortative mixing by age [28]. The lack of significant disassortative mixing by age in association with HPV detection in our sample conforms with research that showed HPV infection in men was not related to age [8]. HPV might be a less “discriminating” virus than other STIs and HIV due to high prevalence, lack of symptoms, and less immediate need for treatment.

Bridging is a concept that has been addressed in several ways in the literature of STI research. Bridging populations contribute to the spread of STI to the general population; without the bridging population, the infection would have been contained in the high-risk core population [29]. The position in the sexual network of the bridging population versus an individual’s own high-risk behavior may be a key determinant for an individual’s STI status. However, bridging has not been measured uniformly in the literature although it has been mostly based on the structural hole concept [27, 30–32]. One study examining bridging in a sample of young MSM in association with HIV status was conducted using a snowballing method and an algorithm to match participants on a connected network [33]. In such method and context, MSM located in the bridging position were associated with being HIV infected [33]. Two studies focused on measuring bridging at the community and neighborhood levels [30, 31].

In a network formed by communities as network nodes and a network connection formed by two individuals from different neighborhoods forming a sexual relationship, the community sexual bridging can be defined in various domains, such as walk-betweeness or flow-betweeness [30]. Certain communities may exhibit high walk-betweeness scores but low scores in other bridging measures [30], showing a context-specific need to discuss the association between the level of bridging and STI prevalence. Due to the wide range of operation of the bridging concept in STI research, the findings should be examined with the definition and context of bridging in mind. Findings from our purely egocentric approach should not be directly compared to other studies with different design. However, our study provides other evidence for the role of bridging in disease transmission, using an egocentric network collection approach.

Moreover, given the high prevalence of online dating in MSM in Taiwan [34], it may increase the likelihood that an individual’s sex partner had not had sex with another partner if both partners were sought via the Internet or apps. Chances for sex partners knowing each other was low and may result a low bridging score. The egocentric network of our sample may be a nondense network, an open, or a radial network where very few alters know each other [35]. The association between low bridging scores and a higher likelihood for HPV detection is in line with a study that compared virtual and physical space to seek sex partners for MSM in East and South-East Asia [36], indicating that greater risk exists among those who use both channels to seek sex partners.

Study findings should be interpreted in the context of the following limitations. First, the study utilized only cross-sectional data; hence, we can only identify HPV detection, and not account for chronical, recurrent, or definite infection. Temporal order cannot be fully established, especially regarding when participants were first exposed to HPV. Second, the majority of the study sample were recruited from a community-based NGO. Study findings may not be generalized to other populations, such as a sample with a higher number of sex partners or a higher prevalence of HIV and other STIs. Third, the egocentric network approach provides the benefit of having detailed information for each partner, yet, due to the burden in recalling too many partners, participants can only report sex partners up to five. It provides a snapshot of sex life, but may not be fully representative of a complete history. Fourth, because HPV screening is not part of a routine STI checkup, it is rare for MSM to know whether their male sex partners have HPV infection. Thus, we did not ask participants to self-report whether their partner had HPV infection. Our study also did not provide HPV screenings for sex partners. Considering that anogenital HPV infection is spread through sexual practices between partners, a lack of information on partners’ HPV status is a major overlook for network factors related to HPV detection.

Our study demonstrated that an individual’s sexual network can be associated with his HPV status, although not all of the network factors were consistent with those associated with HIV or other STIs. An assessment of sexual network characteristics in association with HPV infection can advance our understanding of HPV transmission patterns. Our study results may help related authorities evaluate the current policies for HPV vaccination and reconsider the needs of this vaccination-targeted population. Our findings can help inform the design of targeted prevention and intervention programs for future studies.

## Supporting information

S1 Table Dataset used for the paper (ego). (CSV)

S2 Table Dataset used for the paper (alter). (CSV)

S3 Table Codebook. (XLSX)
